# Accuracy of Lung Ultrasound Compared to Chest Radiography for Diagnosing Pneumonia in Children: A Hospital-Based Cross-Sectional Study

**DOI:** 10.4314/ejhs.v34i1.8S

**Published:** 2024-10

**Authors:** Leul Adane Chemeda, Daniel Zewdneh Solomon, Hanna Damtew Taddese, Henok Hailemichael

**Affiliations:** 1 Pediatric Radiology Fellow, Addis Ababa University, College of Health Sciences, Department of Radiology; 2 Pediatric Radiologist, Addis Ababa University, College of Health Sciences, Department of Radiology; 3 Cardiothoracic Imaging Fellow, Addis Ababa University, College of Health Sciences, Department of Radiology; 4 Radiologist, Wolaita Sodo University Hospital

**Keywords:** Lung ultrasound, chest radiography, CT scan, pneumonia, children, consolidation, interstitial pattern

## Abstract

**Background:**

Traditionally, pediatric pneumonia is diagnosed through clinical examination and chest radiography (CXR), with computed tomography (CT) reserved for complications. Lung ultrasound (LUS) has gained popularity due to its portability and absence of ionizing radiation. This study evaluates LUS's accuracy compared to CXR in diagnosing pneumonia in children.

**Methods:**

We conducted a cross-sectional study from April to September 2023 involving 108 children aged 14 or younger admitted with pneumonia. Each child underwent LUS using a 5-zone scanning protocol, followed by CXR, with the latter interpreted independently by two consultant radiologists. Agreement between LUS and CXR for diagnosing consolidation and interstitial pneumonia patterns was assessed using Cohen's Kappa (k) with SPSS version 26.0.

**Results:**

Pneumonia was radiographically confirmed in 79 children (73.1%). LUS detected consolidation in 41.7% of cases, compared to 43.5% for CXR, demonstrating a sensitivity of 97.8%, specificity of 95.2%, positive predictive value (PPV) of 93.6%, and negative predictive value (NPV) of 98.4%. LUS showed higher accuracy for interstitial lung patterns (sensitivity 93.6%, specificity 97.4%). The agreement between LUS and CXR was excellent, with Cohen's Kappa values of 0.908 for consolidation and 0.863 for interstitial pneumonia. LUS also identified more pleural effusions (11.1%) compared to CXR (6.5%).

**Conclusion:**

LUS demonstrates comparable diagnostic accuracy to CXR for pneumonia, exhibiting high sensitivity and specificity for pneumonia-related features. It outperforms CXR in detecting small-volume consolidations and effusions, supporting its routine use in clinical settings.

## Introduction

Effective management and prevention of pneumonia-related complications depend on early diagnosis. The traditional approach to diagnosing pneumonia includes clinical history, physical examination, and laboratory tests. However, clinical examination alone lacks sufficient reliability (1,2).

Chest infections are primarily diagnosed through clinical assessment, supported by plain chest radiography (CXR) as the first-line imaging modality, while computed tomography (CT) is reserved for complications. Unfortunately, access to CXR and CT may be limited in developing countries, making ultrasound a viable alternative (3).

Lung ultrasound (LUS) is not yet a standard diagnostic tool for pneumonia. However, it offers advantages over CXR and CT, including affordability, portability, and safety (4). Research indicates that LUS may have higher sensitivity and specificity for diagnosing pediatric community-acquired pneumonia (5). Given these advantages, LUS is a promising candidate to replace CXR as the primary imaging tool for suspected pneumonia.

Globally, pneumonia accounts for 14% of fatalities in children under five, with a significant burden in Ethiopia, where approximately 4 million cases occur annually (6, 7). Tikur Anbessa Specialty Hospital (TASH) reports severe pneumonia in 88.9% of pediatric hospitalizations and was the primary cause of death (8, 9). Current diagnostic practices heavily rely on clinical history and examination, but these methods often fall short (3, 10).

CXR remains the preferred imaging modality, useful for management of community-acquired pneumonia and related complications. However, access to radiographic imaging services is limited, affecting diagnosis and leading to increased antibiotic resistance due to overprescription (11, 12, 13).

LUS offers a simple, safe, and cost-effective diagnostic alternative with high sensitivity and specificity (14, 15). Despite evidence supporting LUS, no studies have assessed its efficacy in diagnosing pneumonia in Ethiopian children aged over five years. This study aims to compare the accuracy of LUS with CXR in children under 14.

## Materials and Methods

**Study area and period**: The study was conducted at Tikur Anbessa Specialized Hospital (TASH) in Addis Ababa, Ethiopia, from April to September 2023. TASH serves approximately 470,000 to 500,000 patients annually, with a significant pediatric population.

**Study design**: This was a hospital-based cross-sectional study.

**Study population**: Participants included pediatric patients (aged 14 and below) with clinically suspected pneumonia presenting to TASH's radiology department.

**Sample size determination**: The sample size was calculated using the Daniel formula, leading to a total of 108 participants.

**Sampling method**: Consecutive sampling was employed for all patients presenting to the radiology department until the desired sample size was reached.

### Eligibility

*Inclusion criteria:* Clinically suspected pneumonia diagnosed at TASH radiology department

Children aged 14 years or younger, consent obtained from parents/guardians

*Exclusion criteria:* More than 24 hours between LUS and CXR, known radiographic results prior to the investigation, non-diagnostic CXR results, lack of consent from parents/guardians

### Imaging protocols

*CXR:* Patients underwent either posterior-anterior or anteroposterior CXR, interpreted by two independent consultant radiologists.

*LUS Procedure:* LUS was performed by a trained consultant radiologist unaware of CXR results. Equipment used included a Mindray DC70 ultrasound machine with a 6-13 MHz linear probe.

**Ethical considerations**: Ethical approval was granted by the Institutional Review Board (IRB) of Addis Ababa University. Informed consent was obtained from participants and/or guardians.

**Statistical analysis**: Data were entered into SPSS version 26.0 to calculate sensitivity, specificity, and predictive values, with Cohen's Kappa (16) used to assess agreement between LUS and CXR.

## Results

Among 108 participants, 65 were male (60.2%) and 43 female (39.8%). The age range was 2 to 158 months, with a mean of 48.4 months (Table 1).

**Table 1:** Baseline Characteristics of Patients (N=108)

Patient characteristics	Frequency	Percent
**Demographic data**		
Male	65	60.2
Female	43	39.8
**Clinical History**		
Cough	93	86.1
Fever	88	81.5
Difficulty breathing	61	56.5
**Parental Education**		
Primary	63	58.3
Secondary	28	25.9
Tertiary	17	15.8
**Area of Residence**		
Urban	77	71.3
Rural	31	28.7

Pneumonia was confirmed in 79 children (73.1%) using CXR as reference standard. LUS showed a sensitivity of 96%, specificity of 81.2%, PPV of 92.4%, NPV of 89.7%, and overall diagnostic accuracy of 94.1% (Table 2).

**Table 2:** Classification of pneumonia patient by LUS using CXR as a reference Standard

CXR	LUS			Sensitivity	Specificity	PPV	NPV

	Positive	Negative					
Positive	73	6	79	96.0%	81.2%	92.4%	89.7%
Negative	3	26	29				
Total	76	32	108				

LUS revealed abnormalities consistent with pneumonia in 76 participants, including pleural effusions and atelectasis. (Table 3) LUS identified 45 patients with pneumonic consolidations, including small consolidations undetected by CXR.

**Table 3:** Prevalence of CXR and LUS Findings (N=108)

CXR findings	Frequency	Percent
Normal	28	25.9
Consolidation	47	43.5
Interstitial pattern	32	29.6
Pleural effusion	7	6.5
Atelectasis	4	3.7
**LUS findings**		
Normal	26	24.1
Consolidation	45	41.7
Size ≥ 1cm	36	80
Size < 1cm	9	20
With air bronchogram	40	88.9
Without air bronchogram	5	11.1
Interstitial pattern	31	28.7
Pleural effusion	12	11.1
Atelectasis	7	6.5

**Diagnostic performance**: LUS demonstrated a sensitivity of 97.78% and specificity of 95.24% for consolidation, with a high agreement with CXR (Cohen's Kappa = 0.908). For interstitial patterns, LUS sensitivity was 93.55% and specificity 97.4% (Cohen's Kappa = 0.863) (Table 4).

**Table 4:** Comparison of CXR and LUS in the Diagnosis of Consolidation and Interstitial Pneumonia

			LUS		Total	Sensitivity	Specificity	PPV	NPV

			Positive	Negative					
**Consolidation**	**CXR**	Positive	44	**3**	47	97.78%	95.24%	93.62%	98.41%
		Negative	**1**	60	61				
		Total	45	63	108				
**Interstitial**		Positive	**29**	**3**	32	93.55%	97.4%	90.63%	97.37%
**Pneumonia**		Negative	**2**	**74**	76				
		Total	31	77	108				

## Discussion

Pneumonia remains the single largest infectious cause of childhood mortality worldwide, disproportionately affecting children in low- and middle-income countries. Early diagnosis and treatment of pneumonia is critical for reducing morbidity and mortality. This study aimed to evaluate the diagnostic accuracy of LUS compared to CXR for pneumonia diagnosis in children.

The prevalence of radiographic pneumonia in this study was 73.1%. This is high compared to the 24.9% and 18% prevalence reported by Shah et al and Rees CA et al (3, 17). The discrepancy likely arises from our inclusion of interstitial lung patterns in the definition of pneumonia, in addition to lung consolidation and pleural effusion, as used by Rees CA et al. Viral pneumonia commonly manifests as interstitial changes on imaging. The inclusion of these interstitial pneumonias increased the prevalence of pneumonia in our study.

Lung consolidations were identified in 41.7% of patients on LUS and 43.5% on CXR. For the detection of lung consolidation, LUS demonstrated excellent diagnostic accuracy, with a sensitivity of 97.8% and specificity of 95.2% using CXR as the reference standard. The high sensitivity enables the LUS to reliably rule out consolidation with a negative predictive value of 98.4%. This finding mirrors the result of a systematic review by Balk et al that reported a LUS sensitivity of 95.5% and specificity of 95.3% for pediatric pneumonia (5).

A key advantage of LUS over CXR is its ability to identify smaller (less than a centimeter) consolidations. In this study, 20% of the consolidations detected on LUS were less than 1 cm in size and were likely missed on CXR. Shah et al found that LUS could identify pneumonic consolidations as small as 0.5 cm compared to the 1 cm limit for CXR (3). The higher resolution of LUS facilitates earlier diagnosis of pneumonia before it becomes radiologically evident, allowing prompt treatment.

The 95.2% specificity of LUS for consolidation in our study was comparable to reported by Long L et al. The study also state that the specificity of LUS for acute pneumonia diagnosis is 100%. This is due to utilization of CT scan as a reference standard, which is more sensitive in detecting small volume consolidation possibly visualized on LUS but not CXR (18).

For interstitial lung patterns, LUS showed excellent diagnostic accuracy with 93.6% sensitivity and 97.4% specificity. However, LUS did not detect central perihilar opacities evident on CXR. The ultrasound waves used in LUS do not penetrate air-filled lungs, which limits its general utility (19).

An important additional advantage of LUS over CXR is its ability to detect pleural effusions. LUS identified effusions in 11.1% of patients compared with only 6.5% on CXR. Studies have shown that LUS can detect pleural fluid volumes as low as 5 ml compared to the 150 ml required for identification on CXR (20). This approach facilitates early diagnosis and treatment of parapneumonic effusions.

A major benefit of LUS is the absence of ionizing radiation exposure, which is an important consideration in children who are more radiosensitive with a longer lifetime risk for radiation-induced malignancies (21). LUS is a safe alternative to serial CXRs for pneumonia surveillance and follow-up.

An inherent limitation of LUS is operator dependency, where accuracy varies based on user experience and training. Formal training in LUS image acquisition and interpretation is essential for clinical use. Simulation-based training models are effective for acquiring competency in LUS skills (22).

The limitation of this study was that we used a consecutive sampling technique which might have a potential selection bias, and the exclusion criteria that set no greater than 24hr between LUS and CXR procedures to control the pathophysiologic variabilities. Moreover, it was a single centre study and the lung ultrasound scan was relied on a single radiologist with specialized LUS training. We used CXR as the reference standard, although its sensitivity is lower than CT scan which is considered the gold standard for lung imaging. However, CT is not justified for children as it imposes to higher radiation risks.

Future studies with a larger sample size and random sampling technique is recommended to overcome the limitations in this study. Furthermore, including a control group in future studies to assess the specificity of LUS in distinguishing between pneumonia and other noninfectious respiratory conditions might have a potential benefits; which provide a more comprehensive understanding of LUS's diagnostic performance.

In conclusion, LUS offers comparable or superior diagnostic accuracy to CXR for pneumonia in children, especially for small-volume consolidations and pleural effusions. Its integration into pneumonia diagnosis protocols could significantly improve early diagnosis and treatment, particularly in resource-limited settings. We recommend incorporating Lung Ultrasound (LUS) into pneumonia diagnosis protocols to enhance early diagnosis and treatment in resource-limited settings. LUS can complement Chest X-rays (CXR) for detecting consolidation and interstitial changes, and it may serve as a replacement for CXR in identifying small-volume consolidation and pleural effusion.
